# Experimental Paradigm for the Assessment of the Non-pharmacological Mechanism of Action in Medical Device Classification: The Example of Glycerine as Laxative

**DOI:** 10.3389/fphar.2018.01410

**Published:** 2018-12-07

**Authors:** Claudia Sardi, Stefano Garetto, Laura Capone, Valentina Galbiati, Marco Racchi, Stefano Govoni, Emiliano Giovagnoni, Jacopo Lucci

**Affiliations:** ^1^Natural Bio-Medicine S.p.A, Arezzo, Italy; ^2^Aboca S.p.A, Società Agricola, Arezzo, Italy; ^3^Department of Environmental Science and Policies, Università degli Studi di Milan, Milan, Italy; ^4^Dipartimento di Scienze del Farmaco, Università degli Studi di Pavia, Pavia, Italy

**Keywords:** medical device, glycerine, lubiprostone, osmosis, laxatives, mechanism of action

## Abstract

The evolution of medical devices has led to the introduction of medical devices that include “substances” and which, due to their presentation and sites of application may resemble medicinal products. The difference between substance-based medical devices and medicinal products lies in the proper definition of the principal mechanism of action. The major problem at the moment is the lack of a proper procedure for the demonstration of a mechanism that is “not pharmacological, immunological or metabolic.” We aimed to design an experimental set up to demonstrate the difference between the mechanism of action of two substances used commonly for the treatment of constipation, lubiprostone (example of medicinal product) and glycerine (example of medical device). By implementing cellular models and molecular analyses we demonstrate the difference in their mechanism of action. This set up can be considered an example on the possibility to define a paradigm for the case by case study of the mechanism of action of substances and combination of substances in medical devices.

## Introduction

Medical devices are a wide category of products that are becoming increasingly important in the healthcare system (Racchi et al., 2016). Their evolution has led to the introduction of medical devices that include “substances” and which, due to their presentation and sites of application may resemble medicinal products. Regulation 2017/745 explicitly addresses “medical devices composed of substances or combination of substances” in many paragraphs and in a specific classification rule (Rule 21). This, in comparison with Directive 93/42/EEC is an important acknowledgment of the formerly incorrectly called “borderline devices,” now correctly called “medical devices made of substances.” Borderline products exist until their mechanism of action is identified, on a case by case basis, as explicitly required by Regulation 2017/745 (Reg 2017/745 whereas n.8) (Food Drug Administration, 2005; Alexander et al., 2013). If the specific substance or combination of substances in question are not sufficiently well described in literature or if controversy arises, experimental data has a fundamental role in such assessment. As soon as the mechanism of action is determined, a product made of substances or combination of substances ceases to be borderline and is recognized as either a medical device or a medicinal product. The difference between a medical devices made of substances and medicinal products lies in the principal mechanism of action with which they achieve the therapeutic effect, which also determines their regulatory classification. A medical device has a “non-pharmacological, immunological or metabolic” mechanism of action while a medicinal product has a “pharmacological, immunological or metabolic” mode of action. When the nature of a mechanism of action is not intuitive or not known, it should be demonstrated experimentally. Comparisons may be useful. One significant example is the correct classification of glycerine suppositories or enemas for the treatment of constipation. Constipation is defined as unsatisfactory defecation characterized by infrequent stools, difficult stool passage or both. The treatment of constipation includes laxatives that can act by different mechanisms. One of the major mechanism of action of laxatives includes enhancement of fluid retention by establishing an osmotic gradient at the site of action (Harris, 2005; Andrews and Storr, 2011; Portalatin and Winstead, 2012). Osmosis is a physical process where a solvent moves across a semi permeable membrane (permeable to the solvent, but not to the solute) separating two solutions of different concentrations. The pressure driving this movement (osmotic pressure) does not depend on solute identity (Kii, 1989; Hammel and Schlegel, 2005; McQuarrie et al., 2011). Aqueous hypertonic or hypotonic solutions can be established on either sides of the semi permeable membrane of a cell, leading to movements of water which represents the solvent. Cell volume is proportional to cell water content. In the rectum, greater volumes of stools increase distension and a conscious urge to evacuate, and favors the peristaltic reflex (Andrews and Storr, 2011). Yet, not all substances create a water movement in the rectum by similar means, even if the therapeutic effect is the same (evacuation) as well as the intermediate biological effect (establishing a flux of water molecules at the site of action of the substance). Therefore, it is clear that therapeutic effect and mechanism of action are two distinct concepts, which have been specifically defined previously (Racchi et al., 2016). The aim of this paper is to verify whether specifically designed tests can identify different mechanisms of action of two substances having the same therapeutic effect, and provide any evidence lacking in the literature. The two substances studied are glycerine and lubiprostone. Both create a movement of water molecules toward the gut lumen at their site of action and are used as laxatives. However, they act via a very different mechanisms (Lacy and Levy, 2008; Sweetman, 2009). Glycerine in high concentrations/quantities is usually applied locally and works by directly establishing a hyperosmotic environment at the site of action. Lubiprostone is given by oral administration in micrograms daily. It is a bicyclic fatty acid that activates type-2 chloride channel (ClC-2) in the gastrointestinal tract, increasing chloride concentration in colon fluid with associated passive transport of sodium across the mucosa, thus generating a water movement toward the lumen of the intestine. The action of both products increases fluid presence into the colon lumen which promotes peristaltic waves, thus improving symptoms of constipation. Here we provide a methodological experimental pharmacology set up to demonstrate the different mechanism of action of these two substances. We examine the effects of short-term lubiprostone and glycerine treatments on cell morphology and on cAMP second messenger signaling in two specific cellular models, human colonic adenocarcinoma cell line (T84) and human dermal fibroblasts (HuDe), to demonstrate that the mechanism of action of lubiprostone is dependent on the presence of specific biological targets while that of glycerine is not. We know that glycerine acts as an osmotic agent, therefore lacking any pharmacological specificity. The experimental set up presented in this paper can be considered an example on the possibility to define a paradigm for the case by case study of the mechanism of action of substances and combination of substances in medical devices.

## Materials and Methods

### Cell Culture

Glycerine and lubiprostone are approved medications to manage chronic constipation. The gastrointestinal effects of lubiprostone appear to be mediated by increased Cl^−^ secretion across the apical membrane via specific chloride channels. Considering several lines of evidence linking lubiprostone to the activation of ClC-2 channel in T84 cell line, we chose this colonic adenocarcinoma cell line as a model of intestinal epithelial cells relevant for the dissection of the mechanism of action considered in the study (Barrett and Keely, 2000; Bali et al., 2001; Cuppoletti et al., 2004; Ao et al., 2011; Jin and Blikslager, 2015). The T84 colonic adenocarcinoma cell line was purchased from American Type Culture Collection (ATCC) (Manassas,VA, USA). Cells were grown in DMEM/F12 medium supplemented with 5% heat-inactivated fetal bovine serum (FBS), penicillin (100 U/ml), streptomycin (100 μg/ml). To gain further insight on the mechanism of action of the compounds tested, we referred to a model lacking the ClC-2 channel. We confirmed via reverse transcription quantitative polymerase chain reaction (RT-qPCR) that, human dermal fibroblasts (HuDe) cells do not express the considered chloride channel and were therefore chosen as an experimental counterpart of T84 cells. The HuDe cell line was obtained from Istituto Zooprofilattico Sperimentale della Lombardia e dell'Emilia Romagna (Brescia, Italy). Cells were amplified in MEM containing 10% heat-inactivated Fetal Bovine Serum, sodium pyruvate (1%), streptomycin (100 μg/mL) and penicillin (100 U/mL). All media and supplements were purchased from Thermo Fisher Scientific (Walthan, MA, USA). Both cell lines were incubated at 37°C, in a humidified atmosphere enriched with 5% CO_2_.

### Cell Treatment and Cytofluorometric Analysis

T84 and HuDe cells were seeded on 48 well plate and cultured until 90% of confluence. The cells were incubated in complete media with clinically effective concentrations of glycerol (G5516, Sigma) or lubiprostone (ab145661, Abcam) for 15 min in a humidified incubator at 37°C, in an atmosphere enriched with 5% CO_2_. Immediately after treatment cells were washed with PBS and stained with Propidium Iodide according to the manufacturer's instructions. Cells were acquired by NovoCyte (Acea) and analyzed by FCS Express 6 (De Novo Software). Forward scatter signal intensity (FSC-H) was used to evaluate changes in the size of cells.

### RNA Extraction and Real-Time PCR

The same number of HuDe and T84 cells were lysed by adding RLT PLUS buffer (QIAGEN) for RNA extraction using a QIA Symphony RNA Kit (QIAGEN). RNA purification was performed according to the manufacturer's instructions. The RNA was reverse transcribed using iScript™ cDNA Synthesis Kit (BIO-RAD). The cDNA was used as a template for quantitative PCR (qPCR) performed with TAQMAN^®;^ Fast Universal Master Mix (Life Technologies). Estimation of the change in gene expression was made using the ΔΔCT method. The following TaqMan gene expression assays were used: GAPDH (Hs02786624_g1), Tubulin (Hs00742828_s1), Actin (Hs01060665_g1), HPRT1 (Hs02800695_m1),ClC-2 (Hs00189078_m1). Raw data were normalized with respect to expression values of the housekeeping genes GAPDH, HPRT-1, Actin and Tubulin. Results were analyzed using Graph Pad PRISM 6.0.

### Intracellular cAMP (cAMPi) Measurement

Cyclic AMP XP Chemioluminescent Assay Kit (Cell Signaling Technology, Danvers, MA, USA) was used to measure intracellular cAMP according to the manufacturer's instructions. T84 and HuDe cells were grown in 96-well plates until they reached 90% confluence. Fresh media with known clinically effective concentrations of glycerol and lubiprostone, Forskolin 10 μM (Sigma F6886), 3-isobutyl-1-methylxanthine 100 nM (IBMX) (I5879, Sigma), solvent (DMSO 0.002%) were added to the wells. Cell lysis and preparation for enzyme immuno-assay were performed according to the manufacturer's instructions. The luminescence was measured using Varioskan LUX microplate reader (Thermo Fisher Scientific, USA). Relative light units (RLU) measured were then interpolate in standard curve obtaining cAMP concentrations.

### Osmotic Gradient

Osmolarity of the glycerine and lubiprostone treatment solutions were measured by osmometer type 15 (LÖSER, Berlin-Spandau, Germany) and concentration titration curve of glycerine and lubiprostone solutions were calculated using Graph Pad PRISM 6.0.

### Statistical Analysis

Statistical analysis was performed in GraphPad Prism. Statistical significance was tested using unpaired *t*-test and one-way ANOVA with Dunnett post-test. Data were presented as mean ± standard deviation.

## Results

For the treatment of cellular models we calculated the optimal and clinically relevant concentrations for both glycerine and lubiprostone, selected based on clinical dosing regimens and concentrations used in previously published experimental studies (Brunton et al., 2006; Camilleri et al., 2006; Park et al., 2017). Glycerine is clinically administered as a laxative at a single dose of 2,250 g (enemas or suppositories). Considering the volume of fluids contained in the rectal ampoule is on average 13.8 ml (Fukudo et al., 2011), the final concentration of the applied solution is usually 1.7 M. The recommended dose of lubiprostone is 24 mcg twice daily (capsules for oral administration). Since the drug acts locally and assuming the volume of the colon as approximately 1.5 L (Andrews and Storr, 2011) the clinically relevant concentration range is estimated at 125–250 nM.

### Effects on T84 Cell Size

**Figure 1:**
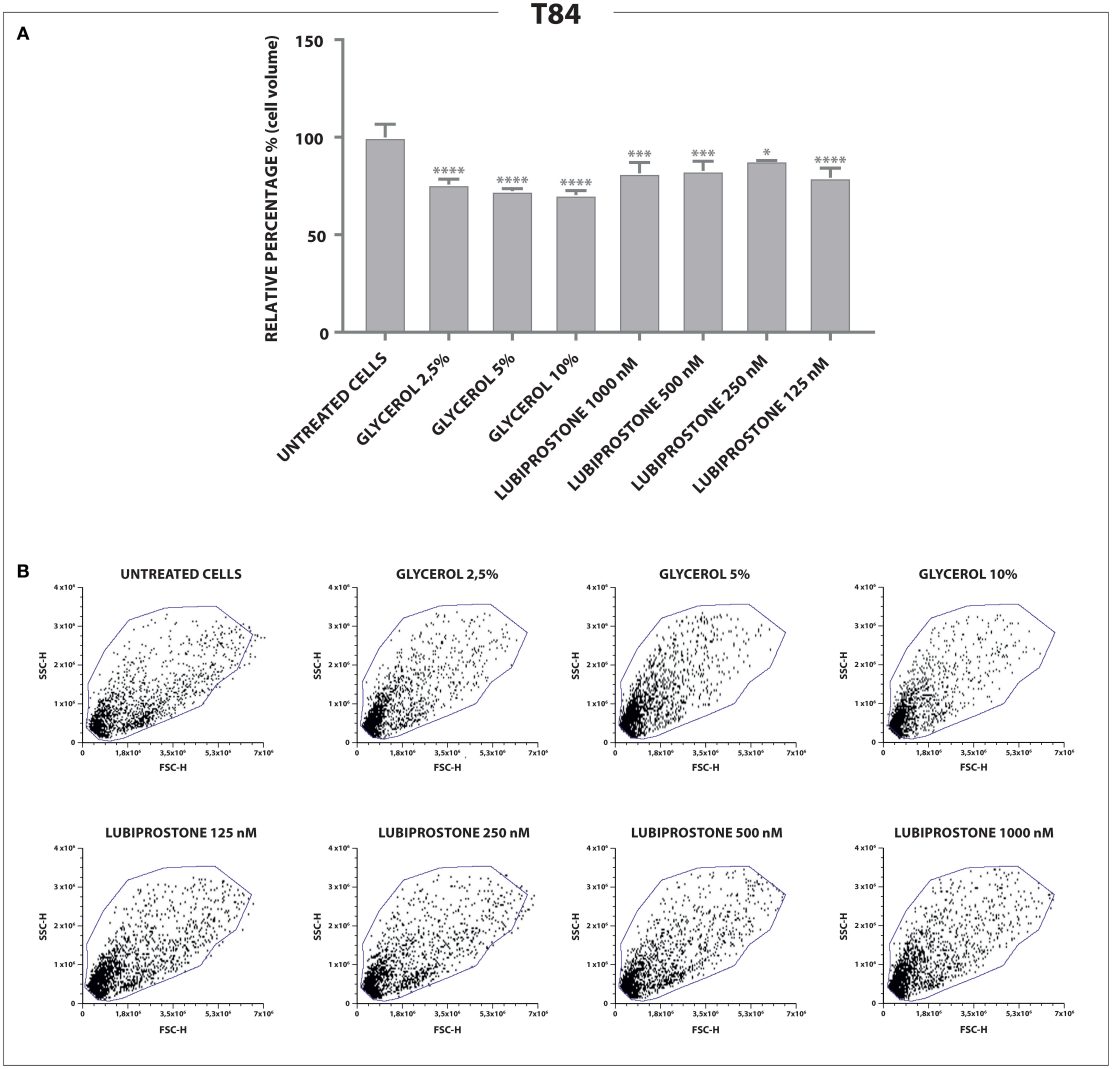
Effects of glycerine and lubiprostone on colonic adenocarcinoma cell line (T84). **(A)** Both lubiprostone and glycerine solutions are able to establish an osmotic gradient that drives an efflux of water from intra to extracellular side in T84 cells, demonstrated with a concomitant reduction of cell size as measured via FSC-H assessment. **(B)** Representative dot plots analyzed by FCS Express 6. (Control[untreated cells], glycerine 2,5%, glycerine 5%, glycerine 10%, lubiprostone 125, 250,500, 1,000 nM). Statistical analysis was performed in GraphPad Prism. Statistical significance was tested using one-way ANOVA with Dunnett post-test. Data were presented as mean ± standard deviation. *p < 0.5; ***p < 0.001; ****p < 0.0001.

Clinically effective dosing regimens were used to investigate the effects of glycerine and lubiprostone on human colon carcinoma cell line T84, widely used as a model system for the study of intestinal ionic transport (Barrett and Keely, 2000; Bali et al., 2001; Fukudo et al., 2011; Jin and Blikslager, 2015). T84 cells were treated with clinically effective concentrations of glycerine and lubiprostone for 15 min. Flow cytometry experiments have been performed to compare whether selected dosing regimens of glycerine and lubiprostone differentially affect cell volume. In this colonic cell line both lubiprostone and glycerine solutions proved to be effective as they are able to establish a water efflux from intra to extracellular side, demonstrated with a concomitant reduction of cell volume as measured via FSC-H assessment (Figure 1).

### Effects on HuDe Cell Size

**Figure 2:**
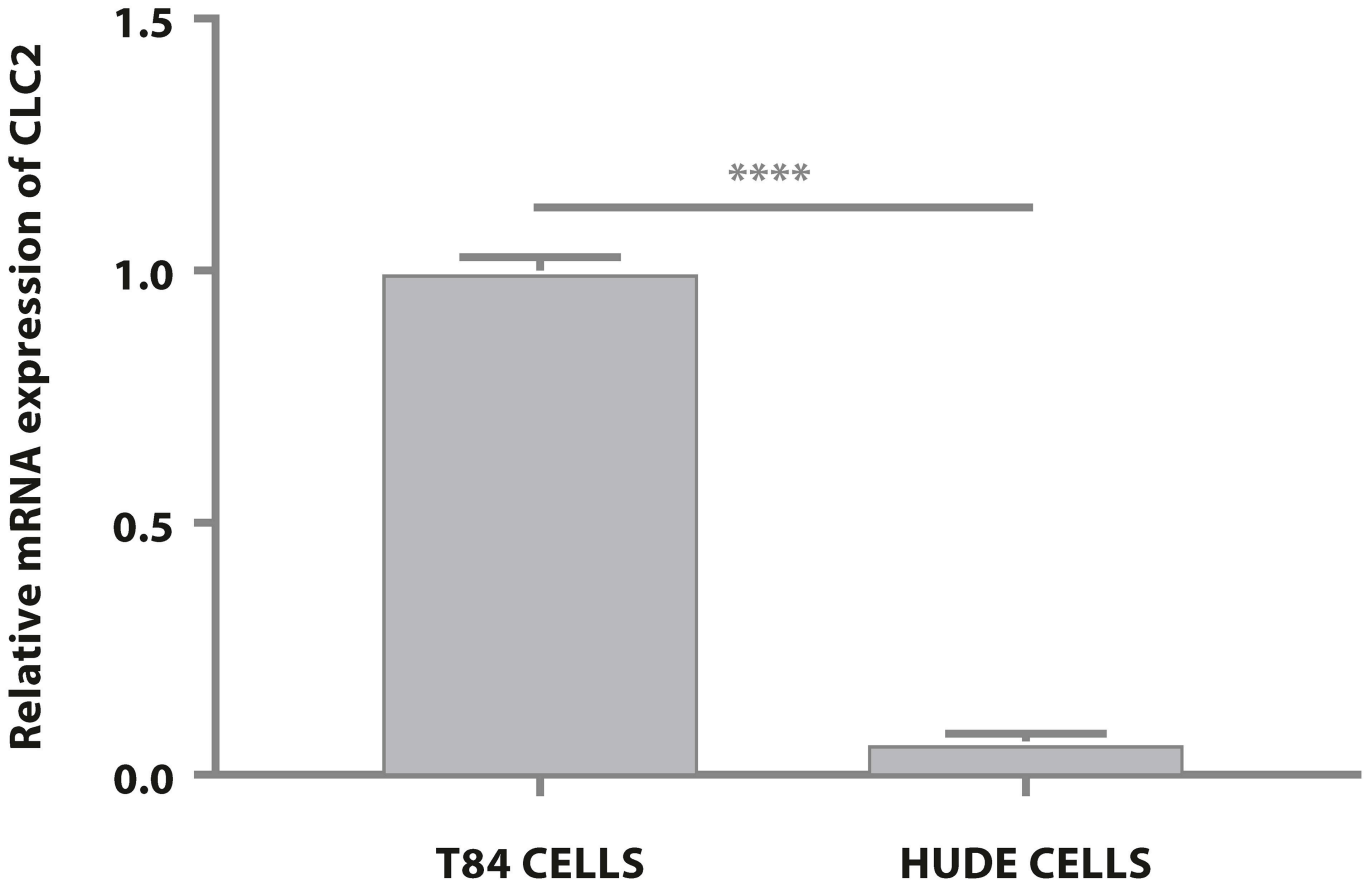
T84 and HuDe gene expression analysis of ClC-2. The expression of ClC-2 occurs only in intestinal cells. Statistical analysis was performed in GraphPad Prism. Statistical significance was tested using unpaired *t*-test. Data were presented as mean ± standard deviation. ****p < 0.0001.

**Figure 3:**
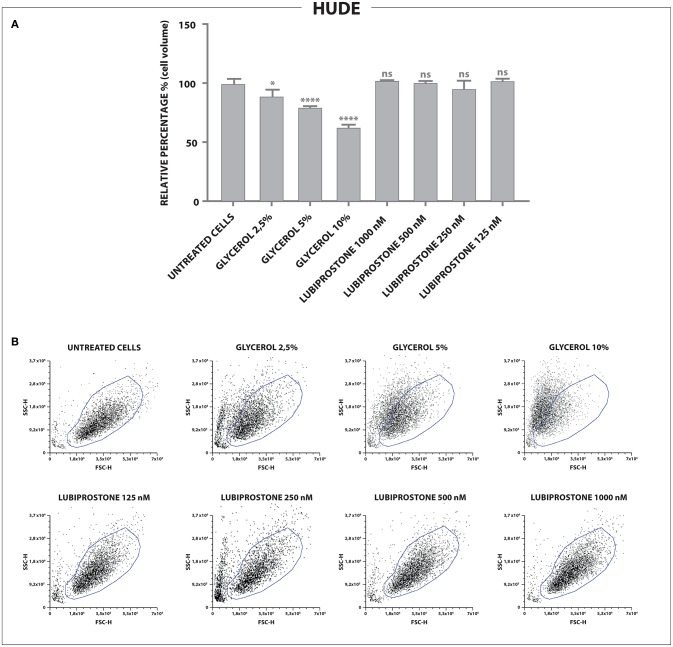
Effects of glycerine and lubiprostone on human dermal fibroblast (HuDe). **(A)** Forward scatter signal intensity (FSC-H) was used to evaluate changes in the size of cells. Lubiprostone was not effective on human dermal fibroblasts. In contrast, all glycerine treatment conditions induced a significant reduction of cell size compared to control cells. (Control[untreated cells], glycerine 2.5%, glycerine 5%, glycerine 10%, lubiprostone 125, 250, 500, 1,000 nM). **(B)** Representative dot plots analyzed by FCS Express 6. (Control[untreated cells], glycerine 2.5%, glycerine 5%, glycerine 10%, lubiprostone 125, 250, 500, 1,000 nM). Statistical significance was tested using one-way ANOVA with Dunnett post-test. Data were presented as mean ± standard deviation. *p < 0.5; ****p < 0.0001.

Having established that both compounds, as expected, modulate water efflux in the T84 colon epithelial model we explored further the different mechanisms of action. Lubiprostone has been reported to increase transepithelial Cl^−^ transport in T84 colonic epithelial cells by activating ClC-2 (Cuppoletti et al., 2004). The ClC-2 chloride channel is a member of the voltage-gated chloride channel family and is localized to the apical cell membrane in human intestine. It is likely that in cells devoid of these channels, lubiprostone cannot exert its effect on water fluxes and therefore will not modify cellular volumes while glycerine, acting through a physical mechanism (giving rise to osmotic pressure) will retain its properties. We used reverse transcription quantitative polymerase chain reaction (RT-qPCR) to examine gene expression of ClC-2 in T84 cells and in human dermal fibroblast (HuDe). We observed that T84 cells express the considered chloride channel (Bali et al., 2001), while HuDe cells do not confirming that the ClC-2 channel is expressed only in colonic cell line (Figure 2). We next examined via flow cytometry the effects of lubiprostone and glycerine in HuDe cells which differ from T84 in terms of ClC-2 expression. Lubiprostone was not effective on HuDe cells in terms of reduction of cell size. In contrast, all glycerine treatment conditions induced a significant reduction of cell size compared to control cells (Figure 3). Independently on the cell type glycerine was effective in promoting the constitution of an osmotic gradient. The presence of ClC-2 only in intestinal cells, strongly suggests that achievement of efficacy by lubiprostone requires the interaction with specific targeted cellular structures, in this case a chloride channel. On the other hand, the unspecificity in the mechanism of glycerol-induced constitution of a hyperosmotic gradient, confirmed its lack of a dependence on tissue specific targeted cellular structure.

### Confirmation of the Signaling Pathway of Lubiprostone Through cAMP Measurements

In light of the differential effect of lubiprostone and glycerine as a function of the presence or absence of a specific pharmacological target we set to further explore the differences in mechanism of action by studying the activation of cellular signaling in the two cellular models. Activation of the cyclic AMP (cAMP) signaling pathway is typical of lubiprostone activity (Ao et al., 2011), therefore T84 and HuDe cells were grown as indicated in the Methods section and treated with clinically relevant concentrations of glycerine, lubiprostone, and forskolin as a receptor-independent activator of adenylyl cyclase to be used as positive control. Isobutylmethylxanthine (IBMX) was used to avoid decrease of signal due to concomitant degradation of cAMP by cellular phosphodiesterases. The metabolic response of the different cell types we interrogated was radically different, despite the establishment of a comparable phenotype at the level of the loss of cell size, derived from establishment of an osmotic gradient. Only ClC-2 positive T84 cells, coupled the loss of cell volume due to the establishment of a lubiprostone activated, Cl^−^ dependent osmotic gradient to the activation of the relevant CIC-2 dependent cAMP pathway. In contrast, while CIC-2 negative HuDe cells still responded to the application of an osmotic gradient due to the administration of a hyperosmotic glycerine solution, they did so without concomitant activation of the very stimulus-unspecific cAMP signaling pathway, arguing in favor of glycerine constituting a hyperosmotic environment in a manner completely independent on cellular structures (Table 1).

**Table 1:** Evaluation of the cAMP signaling independence of glycerine mechanism of action.

**Samples**	**HuDe**		**T84**	
	**cAMP nM**	**Statistical analysis**	**cAMP nM**	**Statistical analysis**
Forskolin 10 μM	0.65	**	1.45	**
Lubiprostone 1,000 nM	0	ns	0.36	**
Lubiprostone 500 nM	0	ns	0.36	**
Lubiprostone 250 nM	0	ns	0.17	*
Lubiprostone 125 nM	0	ns	0.26	*
Glycerol 10%	0	ns	0	ns
Glycerol 5%	0	ns	0	ns
Glycerol 2.5%	0	ns	0	ns

Only ClC-2 positive T84 cells, coupled the loss of cell volume due to the establishment of a lubiprostone activated, Cl^−^ dependent osmotic gradient to the activation of the relevant CIC-2 dependent cAMP pathway. In contrast, while CIC-2 negative HuDe cells still responding to the application of an osmotic gradient due to the administration of a hyperosmotic glycerine solution, they did so without concomitant activation of the very stimulus-unspecific cAMP signaling pathway, arguing in favor of a complete indipendence of glycerine dependent constitution of a hyperosmotic environment on cellular structures. Statistical analysis was performed in GraphPad Prism. Statistical significance was tested using one-way ANOVA with Dunnett post-test.

** *p < 0.01*.

### Measurement of Osmolarity of the Clinically Relevant Treatment Solutions

**Figure 4:**
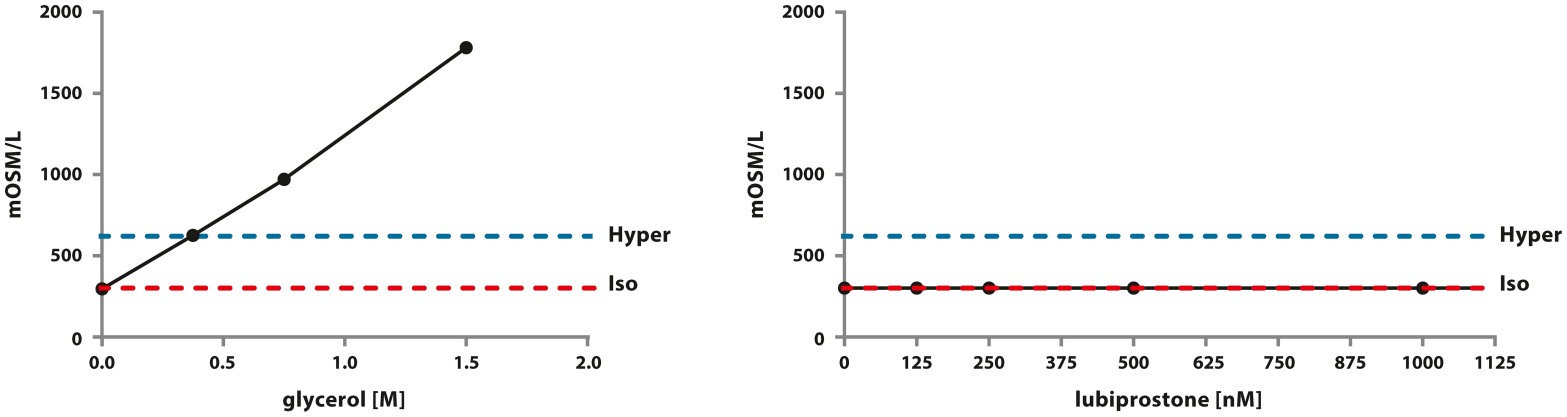
Assessment of osmolarity of glycerine and lubiprostone solutions across abroad concentration range, including clinically relevant ones. The experiment was conducted in an abiotic system using a calibrated osmometer as indicated in the Material and Methods section.

Noticeably, clinically relevant concentrations of glycerine were able to generate a hyperosmotic environment proportionally to its concentration in an abiotic, therefore by default independently from any cellular structures, system. In contrast, in the clinically relevant range of concentrations, lubiprostone was not able to generate a hyperosmotic environment in an abiotic system. Concentrations higher than 1.5 M of glycerine are not assessable via the selected method of investigation as they cannot freeze (Figure 4). These results clearly show that glycerine induces an osmotic change of the microenvironment due to the colligative properties of the solution prepared, without even the need of any biological intermediary. Glycerine acts through a non-pharmacological mechanism of action.

## Discussion

Evolution of medical devices and their regulation has led to the market approval of devices that resemble in form to medicinal products due to the fact that they are made of substances. For this reason they are called “medical devices made of substances.” Within regulatory documents, the separation point between drugs and medical devices lies in the mechanism of action, which in many cases is neither immediate nor intuitive. A clear definition and correct interpretation of “pharmacological, immunological and metabolic mechanism of action” is the most important starting point. For instance, sometimes the concepts of “therapeutic effect” and “mechanism of action” are confused or wrongly considered to be equivalent and there is substantial lack of knowledge as to how to demonstrate experimentally non pharmacological mechanisms of action, which characterize medical devices made of substances. There is no official European definition of the terms “mode of action/mechanism of action” and “effect.” We have previously reported that the mechanism of action of a substance is defined in dictionaries and textbooks as the mechanism by which an active substance produces an effect on a living organism or in a biochemical system. The pharmacological mechanism of action is usually considered to include the identification of specific molecular targets to which a pharmacologically active substance binds and whose biochemical action it modifies (Food Drug Administration, 2005; Alexander et al., 2013; Racchi et al., 2016). The effect is the observable consequence of the action of the substance, independently of the mechanism with which it is achieved. These considerations are confirmed by FDA definition of mode of action as “the means by which a product achieves its intended therapeutic effect or action,” thus well separating the two concepts (mode of action vs. therapeutic effect) (MEDDEV 2. 1/3 rev 3, 2005). Lastly, it must be noted that sometimes, therapeutic effect is also called therapeutic action, adding to the possible confusion with mode of action. Therapeutic effect is not specifically defined by FDA but includes any effect or action of the product intended to diagnose, cure, mitigate, treat, or prevent disease, or affect the structure or any function of the body (Bouin et al., 2001). Relevant to experimental pharmacologists should be the fact that many of the regulatory decisions today are left to the interpretation of the legislator and pharmacological research and proper experimental evidence are sometimes lacking. We therefore engaged into the exercise to suggest an example of experimental set up to demonstrate the difference between “mechanism of action” and “therapeutic effect.” This exercise should represent a reference frame for the future application of experimental paradigms to correctly determine the pharmacological vs. non-pharmacological modes of action in medical device regulation. We used as example two substances for the treatment of a common ailment and that we can demonstrate have the same effect with different mechanisms of action. Specifically one is clearly “pharmacological” in nature while the other relies solely on a “physical” mechanism. Here we present a set of experimental data which allow for an assessment of the mechanism of action of glycerine compared to lubiprostone used as laxatives. A pharmacological experimental set up was designed to discern whether or not water movement is achieved via a pharmacological stimulation of a receptor or non-pharmacological (high osmolarity of substance solution) activity and constitute a support to regulatory decisions, verified by pharmacologists and applied to medical devices. The specific phenomenon induced by laxatives which generate an osmotic gradient in the intestinal lumen is the passage of water passively across epithelial barrier in response to the osmotic gradient established by the presence of osmolytes. Osmosis does not cause an active secretory act of the intestinal epithelium, but a passive passage of water, due to the environmental conditions of the lumen. While doing so, the osmotic pressure induced by glycerine produces a physical stress factor for the intestinal epithelium and mild inflammatory changes occurring in response to osmotic pressure may cause nerve stimulation and contractile reflex (Hardcastle and Mann, 1970). Evoking such reflex does not entail a targeted specific key-lock interaction with a specific receptor. There are no reports of specific glycerine-sensitive receptors on human gastrointestinal mucosa nor on sensory nerve endings within the mucosa itself. It is well known that mechanoceptors or osmoceptors can trigger reflexes such as peristalsis, for example, in case of mechanical distention of the bowels and rectum due to food and feces, respectively (Andrews and Storr, 2011). As a final confirmation, our data show that in an abiotic system it is clear that clinically relevant solutions of glycerine have a distinct osmolarity which is related to their concentration while active lubiprostone solutions are always iso-osmotic at all concentrations. These same concentrations produce a change in cell size as expected from mechanisms that induce water efflux from the cells. The experimental paradigm shows unequivocally the difference in mechanism of action. Lubiprostone leads to the generation of a water efflux through the modification of a biological substrate (ion channel) only in those colon epithelia cellular models (T84 cells), which possess the specific ClC-2 receptor-channel, and induce a signaling response (cAMP production). On the other hand the effect of glycerine on cell size is obtained also in cells devoid of the specific ClC-2 channel (HuDe) and without affecting second messenger suggesting in fact that glycerol is a physical agent (induces an osmotic gradient in the microenvironment). Concerning cAMP production it should be noted that certain cellular structures (Stokes et al., 2004; Yau and Hardie, 2009; Sodhi and Hartwick, 2014) such as some mechanoreceptors, can trigger cAMP dependent pathways in response to non-pharmacological stimuli (i.e., physical, mechanical, chemical). In these cases however the consequence is the activation of a second messenger metabolic pathway which is associated to the effect of the chemical or physical trigger. In the case of a product that achieves its efficacy in the presence of a concomitant accumulation of cAMP or other second messengers, further studies should be performed in order to define the specific triggering event underlying such observation. The fact that glycerine does not induce second messenger accumulation further strengthens the conclusion that its mechanism is not pharmacological. Overall our investigation suggests that current definitions of “pharmacological means” reported on the European Meddev2. 1/3 rev 3 (MEDDEV 2. 1/3 rev 3, 2005) and the more extensive elaboration of these definitions proposed by Racchi et al. (2016) can serve as a grid for experimental pharmacologists to establish the pharmacological/non pharmacological mechanisms of action of a substance or combination of substances. Looking at these definitions and the proposed definitions for chemical and physical mechanisms of action (Racchi et al., 2016) we can verify their adequacy in tracing the correspondence with experimental data (Table 2) and may suggest that from the experimental data, the definitions of pharmacological, chemical and physical modes of action are reliable and determine that while lubiprostone has a pharmacological mode of action, glycerine has a physical mode of action. It is of utmost importance to distinguish between mechanisms of action and effect, because pharmacological and non-pharmacological mechanisms of actions can yield the same biological effect. By evaluating the specific example here reported, we can highlight the differences between the pharmacological mode of action of lubiprostone and the physical mode of action of glycerine: importantly, to the best of our knowledge this is the first time that a pharmacological and a non-pharmacological mode of action of two substances yielding the same effect have been dissected experimentally and compared. Comparing the evidence produced with the definitions of pharmacological and physical and chemical modes of action we can conclude that the definitions fit scientific evidence, confirming that physically active substances target the environment and may require neither cellular systems nor specific receptors.

**Table 2:** Correspondence between experimental data and definitions of pharmacological, chemical and physical modes of action reported in literature (MEDDEV 2. 1/3 rev 3, 2005; Racchi et al., 2016).

**Mechanism of action**	**Distinctive elements**	**Experimental data on lubiprostone**	**Experimental data on glycerine**
“Pharmacological means” (Racchi et al., 2016) is understood as a (TARGETED) interaction between the molecules of the substance in question and a cellular constituent, usually referred to as a receptor, which either results in a direct response, or which blocks the response to another agent. Although not a completely reliable criterion, the presence of a dose–response correlation is indicative of a pharmacological effect	Need for a biotic environment	✔ Evidence: no change in the osmotic pressure of experimentally effective solutions	✖ Evidence: increase in the osmotic pressure of experimentally effective solutions
	Need for a specific receptor	✔ Evidence: no activity in absence of ClC-2 receptor	✖ Evidence: osmotic pressure increase is independent from the type of cell
Chemical mechanism of action (Racchi et al., 2016) is intended as the interaction of a substance with other substances present in the body, such as to transform the initial chemical substances (the reactants) into different chemical compounds (the reaction products). These actions should not include the targeted interaction with a receptor and its signaling pathway.	Interaction between substances to create a new substance (excluding receptors)	✖ No new substance is created	✖ No new substance is created
Physical mechanism of action (Racchi et al., 2016) is intended as the interaction of a substance/material with other substances present in the body, such as solely to transform the surrounding environment/matter.	Change in the targeted environment	✖ Evidence: first action of lubiprostone is on the cell not on the environment.	✔ Evidence: the osmotic pressure of the environment is modified directly by the concentration of dissolved glycerine

## Author Contributions

MR, JL, SGo, LC, and EG contributed to the original idea, designed and coordinated the experiments and wrote the paper. SGa, CS, and VG performed the experiments and the analysis, contributed to the final discussion of the manuscript and wrote, amended the paper.

### Conflict of Interest Statement

CS, SGa, and JL are employed in the regulatory/research division of Natural Bio-Medicine S.p.A. EG and LC are employed in the regulatory/research division of Aboca S.p.A. Società Agricola. The remaining authors declare that the research was conducted in the absence of any commercial or financial relationships that could be construed as a potential conflict of interest.
